# The Seascale cluster: a probable explanation

**DOI:** 10.1038/sj.bjc.6690642

**Published:** 1999-09

**Authors:** Richard Doll

**Affiliations:** ICRF/MRC/BHF Clinical Trial Service Unit and Epidemiological Studies Unit, Radcliffe Infirmary, Harkness Building, Oxford, OX2 6HE, UK


*British Journal of Cancer* (1999) **81**(1), 3–5
© 1999 Cancer Research Campaign
Article no. bjoc.1999.0642

Editorial

### The Seascale cluster: a probable explanation

**Richard Doll**

ICRF/MRC/BHF Clinical Trial Service Unit and Epidemiological Studies Unit, Harkness Building, Radcliffe Infirmary, Oxford OX2 6HE, UK

Seldom, if ever, can so few cases of disease have caused so much work and so much public concern for such a long time as the seven cases of leukaemia that occurred in young people under 25 years of age who lived in Seascale during the period 1955–1983. The expected number cannot be calculated precisely, but the excess under 10 years of age was about tenfold (five against 0.5 expected) and there can be no doubt that this 'Seascale cluster', as it has come to be called, constitutes a most unusual happening.

The cluster was discovered by journalists from Yorkshire Television in 1983 in the course of enquiries into the mortality from cancer near Sellafield and was the subject of a television programme (Urquhart et al, 1984). It led to the appointment of a review committee under Sir Douglas Black, which advised the following year that there was sufficient evidence of an unusual incidence of disease to deserve intensive investigation of its cause (Black, 1984). Many studies have consequently been undertaken over the last 15 years, most of which have included non-Hodgkin's lymphoma (NHL) with lymphoblastic leukaemia as, at young ages, there is no clear biological difference between them. The answer that the Black Committee sought has, however, proved elusive. Now with the most recent report by Dickinson and Parker in this issue (pp. 144–151) it may be thought that the answer has already been found.

The occurrence of a cluster of cancers that are among those most easily produced by ionizing radiation in a village 3 km from Sellafield, the principal nuclear reprocessing plant in the UK, led many people to suspect that it was a direct effect of environmental pollution with radioactive waste and this was the first explanation considered by the Black Committee and the Committee on Medical Aspects of Radiation in the Environment (COMARE) that was set up by the Department of Health to oversee the recommended research and to review its findings. The idea was, however, quickly shown to be untenable (Black, 1984). For knowledge of the discharges from the plant showed that the doses that people were likely to have received were far too small to have caused such a large excess of cases, the maximum estimate of their likely effect being a 15% chance of producing one case (COMARE, 1986). This conclusion was later fortified by measurements of Pu and $^{137}$Cs in the bodies of exposed people, which showed that the models that had been used to estimate the doses people received had, for the most part, overestimated them (Popplewell et al, 1988; Stather et al, 1988). With the further information available 10 years later, COMARE (1996) concluded that the doses Seascale residents received that were attributable to discharges from Sellafield were less than 10% of their total dose and about 200 times too small to account for the observed excess of leukaemia and NHL.

Alternative explanations consequently had to be considered and one was suggested by Gardner et al (1990) as a result of two studies. One was thought to show that an excess risk of leukaemia was seen only in children who were born in Seascale and not in those who went to school there but were born elsewhere (Gardner et al, 1987*a*, 1987*b*) and the other that the risk of the disease in West Cumbria was associated with the fathers' employment at Sellafield before the children were conceived (Gardner et al, 1990). The excess risk with the father's employment was not great (a relative risk of 1.97 based on nine cases), but the risk was greatest with the highest cumulative dose that the father had received (relative risk for 100 mSv or more 6.24, 95% confidence limits 1.51–25.76) and a significant dose–response relationship was observed with the dose estimated to have been received in the 6 months immediately prior to the child's conception, when, for much of the time, the more radio-sensitive spermatids, relevant to the child's conception, would have been exposed.

Gardner et al's (1990) idea was never attractive to radiobiologists who knew that no such effect had been observed in the children of the survivors of the Hiroshima and Nagasaki atomic explosions and that animal experiments did not support the idea that chronic low dose exposure would be much more hazardous than moderate doses given acutely and it was soon dispelled by the results of human studies elsewhere over the next few years.

The evidence has been reviewed by Doll et al (1994), Little et al (1995) and COMARE (1996) and the conclusion reached that the hypothesis that irradiation of the testis caused any detectable risk of leukaemia in subsequent offspring could not be sustained. It did not accord with what was known of radiation genetics or of the hereditability of childhood leukaemia. It was not supported by the relationship observed between men's exposure to ionizing radiation and the risk of leukaemia in their offspring in the survivors of the atomic bomb explosions in Japan or in nuclear workers in Ontario, in Scotland, or in Cumbria other than in Seascale. It made no contribution to another cluster in young persons in Egremont, 7 km north of Sellafield (Wakeford and Parker, 1996) and could not account for the excess recorded near Dounreay in Scotland nor near two nuclear sites in the south of England, nor even for the whole of the cluster observed in Seascale, which as Kinlen (1993) showed was not limited to children born there. In these circumstances, it seemed that the association that was observed by Gardner et al (1990) between paternal irradiation and leukaemia in young people born and resident in the West Cumbrian Health District was most readily explained by chance, a conclusion that has subsequently been supported by the results of two large surveys of the risk of cancer in the offspring of nuclear workers, neither of which has provided any evidence for such an association outside the original confines of the Seascale cluster (Draper et al, 1997; Roman et al, 1999).

What then can be the explanation for the occurrence of the Seascale cluster itself? To some statisticians, cognisant of the many clusters that, like the one in Seascale, had been defined post hoc and had not been found to be attributable to any environmental cause, chance was again a reasonable explanation. This, however, was difficult to accept for the excess observed was so extreme and it was, moreover, accompanied by another localized excess in the neighbourhood of the plant. For when Craft et al (1993) examined the spatial distribution of all cancers in young people in the north of England in the period 1968–1985 for which they had reliable incidence data, they found that the excess of acute lymphatic leukaemia in Seascale was the most marked ($P < 0.001$) and that another of the five most extreme findings occurred in Egremont, a small town 7 km north of Sellafield. To this has been added the discovery of the third small cluster near Dounreay (Heasman et al, 1986) the site of the only other nuclear reprocessing plant in the UK. If this were not evidence enough, a significant excess has continued to be observed in Seascale in the years since the cluster was detected (Draper et al, 1993; COMARE, 1996). The idea that the Seascale cluster could be a chance effect must clearly be abandoned.

Another explanation was suggested by Kinlen as long ago as 1988. He noted that Seascale had most unusual geographical and demographic characteristics, being located in a cul-de-sac in an isolated area with the highest proportion of high social class residents of any rural parish in Britain and near the country's largest rural industrial site, where most male Seascale residents worked and where there were also unusually large numbers of mobile construction workers. He noted, too, that the area where the excess near Dounreay was located had many similar characteristics, for it had occurred in Thurso, a small isolated town that had received a major influx of new residents in the 1950s to serve the needs of the nuclear plant and that a small excess of childhood leukaemia (eight cases against 2.87 expected) had also occurred in the rural part of Scotland with the most comparable population increase to Thurso: namely, Kirkcaldy District in Fife, where a new town, Glenrothes, had been created in 1948. He suggested, therefore, that childhood leukaemia might result from a rare response to a common but unidentified infection and that increased risks would occur when populations were mixed that increased the level of contacts between infected and susceptible individuals, something that was particularly likely to occur when urban populations were mixed with rural populations that were likely to contain a higher proportion of susceptibles, especially when the rural populations were of high socio-economic status.

Kinlen and his colleagues subsequently showed that excess risks occurred in many other situations in which mixing of urban and rural populations had occurred: in rural new towns in Britain, in rural areas with a high concentration of servicemen, in local authority growth areas, in rural parts of Scotland (including the Dounreay area) with high proportions of oil industry workers (who mixed with oil workers from towns but had to return regularly to their homes), in towns with the greatest increase in commuting to work, in rural districts that had received the greatest proportion of evacuees from large centres during the Second World War, and in rural areas containing large construction sites where large numbers of workers from other parts of the country were employed (reviewed in Kinlen, 1995; Kinlen et al, 1995). They showed, moreover, that the continued excess in Seascale over so many years was accompanied by continued construction projects (such as the recent building of THORP) involving by renewed influxes of construction workers (Kinlen et al, 1995).

The findings in these and other studies by other workers (Langford, 1991; Petridou et al, 1996; Stiller and Boyle, 1996; Alexander et al, 1997) make a formidable case. It was dismissed by COMARE (1996), however, as an adequate explanation on the grounds that the hypothesis could not account for such a great increase in risk as that observed in Seascale over a prolonged period. In picking out Seascale and comparing it with the other areas in which population mixing had been found to be associated with an increased risk, the Committee had not, however, been comparing like with like, for Seascale had been stumbled on as a parish with an abnormally high risk and was not comparable to the areas in which population mixing had been studied elsewhere. A similar local investigation could have picked out comparable parishes within all these other areas, as was shown in Kinlen et al's (1997) review. There does not, therefore, seem to be anything qualitatively different between the experience in Seascale and other selected high risk parishes in areas in which there was a high degree of population mixing. There remained the question of whether population mixing could account for the cluster quantitatively.

Dickinson and Parker's paper (pp. 000–000) now provides evidence that allows the quantitative effect to be estimated. They used data for over 100 000 children born to mothers living in Cumbria in the years 1969–1989 and followed them to 15 years of age, identifying those who developed leukaemia or any other type of malignant disease before 1993. Children born to mothers resident in Seascale were excluded, as were all cancers in children under 1 year of age, as they were likely to have different causes from those occurring later (Greaves, 1997). Children with acute lymphatic leukaemia (ALL) were classed separately from other leukaemias and NHL were classed separately from other cancers. When both parents were born outside Cumbria they were classed as 'incomers' and the degree of population mixing in each electoral ward was determined by the proportion of parents who were incomers. Electoral wards were characterized according to the degree of population mixing and six other characteristics (including an indicator of social class, the density of births and geographical isolation). The incidence rates of ALL and NHL were increased to a similar extent when the parents were incomers and both showed an increasing trend with population mixing in the wards in which the children were born, characteristics which distinguished them sharply from other leukaemias and other cancers. The model that Dickinson and Parker derived predicted a substantially increased number of cases in Seascale, not as many as were actually observed, but within the limits that might have been expected to occur by the additional effects of chance, leave alone a more precise model that could have been derived from more complete records or one that measured risk directly rather than the surrogate measure of population mixing.

With Dickinson and Parker's paper, the time may now have come when Kinlen's hypothesis of population mixing as a cause of childhood lymphatic leukaemia can be regarded as established. There remains the biological problem of identifying the causative agent (or agents) which may, however, prove exceptionally difficult, if the agent is a common one and the reaction an exceptional response.

### REFERENCES

Alexander FE, Chan LC, Lam TH, Yeun P, Ha SY, Li CK, Lau YL and Greaves MF (1997) Clustering of childhood leukaemia in Hong Kong: association with the childhood peak and common acute lymphoblastic leukaemia and with population mixing. *Br J Cancer* **75**: 457–463

Black D (1984) *Investigation of the Possible Increased Incidence of Cancer in Cumbria*. HMSO, London

Committee on Medical Aspects of Radiation in the Environment (COMARE) (1986) *First report. The Implications of the New Data on the Releases from Sellafield in the 1950s for the Conclusion of the Report on the Investigation of the Possible Increased Incidence of Cancer in West Cumbria.* HMSO, London

*© 1999 Cancer Research Campaign*

Committee on Medical Aspects of Radiation in the Environment (COMARE) (1996) *Fourth report. The Incidence of cancer and leukaemia in young people in the vicinity of the Sellafield site, West Cumbria: Further studies and an update of the situation since the publication of the report of the Black Advisory Group in 1984*. Department of Health, Wetherby

Craft AW, Parker L, Openshaw S, Charlton M, Newell J, Birch JM and Blair V (1993) Cancer in young people in the north of England, 1968–85: analysis by census wards. *Epidemiol Comm Health* **47**: 109–115

Dickinson H and Parker L (1999) Quantifying the effect of population mixing on childhood leukaemia risk: the Seascale cluster. *Br J Cancer* **81**: 144–151

Doll R, Evans HJ and Darby SC (1994) Paternal exposure not to blame. *Nature* **367**: 678–680

Draper GJ, Stiller CA, Cartwright RA, Craft AW and Vincent TJ (1993) Cancer in Cumbria and in the vicinity of the Sellafield nuclear installation, 1963–90. *Brit Med J* **306**: 89–94

Draper GJ, Little MP, Sorahan T, Kinlen LJ, Bunch KS, Conquest AJ, Kendall GM, Kneale GW, Lancashire RJ, Muirhead CR, O'Connor CH and Vincent TJ (1997) Cancer in offspring of radiation workers: a record linkage study. *Br Med J* **315**: 1181–1188

Gardner MJ, Hall AJ, Downes S and Terrell JD (1987*a*) Follow-up of children born to mothers resident in Seascale, West Cumbria (birth cohort). *Br Med J* **295**: 822–827

Gardner MJ, Hall AJ, Downes S and Terrell JD (1987*b*) Follow-up study of children born elsewhere but attending schools in Seascale, West Cumbria (schools cohort). *Br Med J* **295**: 819–822

Gardner MJ, Snee MP, Hall AJ, Powell CA, Downes S and Terrell JD (1990) Results of case–control study of leukaemia and lymphoma among young people near Sellafield nuclear plant in West Cumbria. *Br Med J* **300**: 423–429

Greaves ML (1997) Aetiology of acute leukaemia. *Lancet* **349**: 344–349

Heasman MA, Kemp IW, Urquhart JD and Black R (1986) Childhood leukaemia in Northern Scotland. *Lancet* **1**: 266

Kinlen L (1988) Evidence for an infective cause of childhood leukaemia: comparison of a Scottish new town with nuclear reprocessing sites in Britain. *Lancet* **2**: 1323–1326

Kinlen LJ (1993) Can paternal preconceptional radiation account for the increase of leukaemia and non-Hodgkin's lymphoma in Seascale? *Br Med J* **306**: 1718–1721

Kinlen LJ (1995) Epidemiological evidence for an infective basis in childhood leukaemia. *Br J Cancer* **71**: 1–5

Kinlen LJ, Dickson M and Stiller CA (1995) Childhood leukaemia and non-Hodgkin's lymphoma near large rural construction sites, with a comparison with Sellafield nuclear site. *Br Med J* **310**: 763–768

Kinlen LJ, Craft AW and Parker L (1997) The excess of childhood leukaemia near Sellafield: a commentary on the Fourth COMARE Report. *J Radiol Prot* **17**: 63–71

Langford I (1991) Childhood leukaemia mortality and population changes in England & Wales 1969–1973. *Soc Sci Med* **33**: 435–440

Little MP, Charles MW and Wakeford R (1995) A review of the risk of leukaemia in relation to parental pre-conception exposure to radiation. *Health Phys* **68**: 299–310

Petridou E, Revinthi K, Alexander F, Haidas S, Koliouskas D, Kosmidas H, Piperopoulou F, Tzortzatou F and Trichopoulos D (1996) Space–time clustering of childhood leukaemia in Greece. Evidence supporting a viral aetiology. *Br J Cancer* **73**: 1278–1283

Popplewell DS, Ham GJ, Dodd NJ and Shuttler SD (1988) Plutonium and caesium-137 in autopsy tissues in Great Britain. *Sci Tot Environ* **70**: 321–334

Roman E, Doyle P, Maconochie N, Davies G, Smith PG and Beral V (1999) Cancer in children of nuclear industry employees: report on children under 25 years of age from nuclear industry family study. *Br Med J* **318**: 1443–1450

Stather JW, Clarke RH and Duncan KP (1988) *The Risk of Childhood Leukaemia Near Nuclear Establishments. NRPB-R2515.* National Radiological Protection Board, Didcot

Stiller CA and Boyle PJ (1996) Effects of population mixing and socioeconomic status in England & Wales, 1979–85, on lymphoblastic leukaemia in children. *Br Med J* **313**: 1297–1300

Urquhart J, Palmer M and Cutler J (1984) Cancer in Cumbria: the Windscale connection. *Lancet* **1**: 217–218

Wakeford R and Parker L (1996) Leukaemia and non-Hodgkin's lymphoma in young persons resident in small areas of West Cumbria in relation to paternal preconceptional irradiation. *Br J Cancer* **73**: 672–679

*© 1999 Cancer Research Campaign*

